# Effectiveness of Desensitization Therapy for Grasses and Dust Mites on Asthma Exacerbations and Respiratory Function During the Allergy Season: A Pilot Study

**DOI:** 10.3390/jcm15031045

**Published:** 2026-01-28

**Authors:** Marco Mari, Giorgio Monteleone, Mariaelisabetta Conte, Paola Confalonieri, Francesco Salton, Caterina Antonaglia, Alessandra Galantino, Nicolò Reccardini, Michael Hughes, Pietro Geri, Umberto Zuccon, Marco Confalonieri, Rossella Cifaldi, Barbara Ruaro

**Affiliations:** 1 Pulmonology Unit, Department of Medical Surgical and Health Sciences, Hospital of Cattinara, University of Trieste, 34149 Trieste, Italy; 2 Respiratory Disease Unit, “Santa Maria degli Angeli” Hospital, 33170 Pordenone, Italy; 3 Department of Pneumology, Ruhrlandklinik, University Hospital Essen, University of Duisburg-Essen, 45147 Essen, Germany; 4 Struttura Complessa di Allergologia e di Immunologia, Azienda Sanitaria Friuli Occidentale (ASFO), Ospedale S. Maria degli Angeli, 33170 Pordenone, Italy; 5 Centre for Musculoskeletal Research, Division of Musculoskeletal and Dermatological Science, School of Biological Sciences, The University of Manchester, Manchester M13 9PT, UK; 6 Department of Rheumatology, Northern Care Alliance NHS Foundation Trust, The University of Manchester, Manchester M13 9PL, UK; 7 NIHR Manchester Biomedical Research Centre, Manchester University NHS Foundation Trust, Manchester M6 8HD, UK

**Keywords:** allergic asthma, allergic rhinitis, allergen-specific immunotherapy, sublingual allergen immunotherapy

## Abstract

**Background/Objectives:** Allergic asthma (AA) is a common chronic respiratory disease characterized by airway inflammation and bronchial hyperreactivity triggered by environmental allergens such as pollen and dust mites. Allergen-specific immunotherapy (AIT), particularly its sublingual formulation (SLIT), is the only treatment capable of modifying the disease’s natural course by targeting IgE-mediated sensitization mechanisms. **Methods:** We analyzed demographic, clinical, functional (FEV_1_, FVC, FEV_1_/FVC), and immunological data (specific IgE for Phl p1, Phl p5, Der p1, Der p2, Der p23), alongside asthma control parameters (ACT score), reliever use, and exacerbation frequency, in patients undergoing SLIT for grass pollen and dust mite allergens. **Results:** After at least twelve months of treatment, we observed significant reductions in exacerbation rate (*p* < 0.01) and reliever use (*p* = 0.0002). These improvements were particularly evident in the subgroup receiving grass pollen SLIT (*p* = 0.03 and *p* = 0.01, respectively). An increase in ACT score was observed but did not reach statistical significance (*p* = 0.07), likely due to already high baseline control. All patients reported improved rhinitis symptoms. Lung function parameters showed no significant changes. SLIT was well tolerated, with no serious adverse events or discontinuations. The subgroup of patients treated with dust mite SLIT was small, limiting the statistical power and generalizability of these findings. Consequently, the dust mite results should be interpreted cautiously and considered exploratory. The more robust and statistically supported findings pertain to the grass pollen SLIT group, reflecting a more consolidated evidence base for this allergen. **Conclusions:** SLIT for grass pollen demonstrated promising, statistically supported benefits in reducing exacerbations and improving disease control, supporting its role as an effective adjunct therapy in AA. While dust mite SLIT also showed positive trends, the limited sample size warrants further investigation to confirm these preliminary findings. Overall, SLIT appears to be a safe and potentially beneficial option for patients with AA and allergic rhinitis, but larger studies are needed to substantiate its efficacy across different allergens.

## 1. Introduction

Asthma is a complex, chronic inflammatory disorder of the airways that represents a significant challenge to global health, affecting more than 300 million individuals worldwide [1]. Its natural course is characterized by persistent inflammation involving the walls of the airways, including the bronchi and mucus-producing glands, which leads to airway narrowing and hyperreactivity. This pathological process manifests clinically through symptoms such as bronchospasm, wheezing, shortness of breath (dyspnea), and variable airflow obstruction, which can fluctuate over time and differ in severity [1,2]. Within the diverse subtypes of asthma, allergic asthma (AA) stands out as the most common form. It is primarily triggered by sensitization to aeroallergens, accounting for approximately 80% of childhood asthma cases and around 50% of adult asthma cases [3]. AA is frequently associated with other atopic conditions, such as eczema (atopic dermatitis) and allergic rhinitis (AR), highlighting the shared immunological mechanisms that underlie these disorders [3]. Allergic rhinitis (AR) is an inflammatory condition affecting the nasal mucosa, characterized by symptoms including sneezing, rhinorrhea (runny nose), nasal congestion, and itching of the nose or eyes [4]. It can be seasonal, often linked to pollen exposure during specific times of the year, or perennial, occurring throughout the year and commonly associated with indoor allergens like dust mites, molds, pet dander, or cockroach allergens [4,5]. Notably, AR may significantly influence the development or worsening of AA, as both conditions share similar pathogenic mechanisms such as immune dysregulation and hypersensitivity reactions [5]. The immunological basis of these atopic disorders involves a skewed T-helper 2 (TH2) immune response and the production of immunoglobulin E (IgE)-mediated type I hypersensitivity. This exaggerated immune response is directed against harmless environmental antigens like pollen, dust mites, animal dander, or molds, leading to allergic sensitization [5,6]. In individuals with genetic predisposition, this process results in the production of allergen-specific IgE antibodies, which bind to the surface of mast cells and basophils. Upon subsequent exposure to the same allergen, cross-linking of these IgE molecules triggers mast cell degranulation, releasing a cascade of inflammatory mediators—including histamine, leukotrienes, and prostaglandins—that mediate immediate allergic symptoms such as sneezing, nasal congestion, urticaria, bronchospasm, and itching. Over time, chronic immune activation contributes to ongoing inflammation within the airways, leading to airway remodeling and persistent respiratory symptoms [4,6]. Among the clinical manifestations, asthma exacerbations are particularly alarming events. These episodes can range from mild, requiring minimal intervention, to severe, life-threatening attacks that necessitate emergency medical attention. Such exacerbations greatly impair patients’ quality of life, increase healthcare utilization, and impose substantial economic burdens associated with asthma management [7]. Therefore, preventing exacerbations remains a central goal in asthma treatment strategies. In the realm of allergic diseases, allergen immunotherapy (AIT)—which includes both subcutaneous (SCIT) and sublingual (SLIT) approaches—has emerged as a promising intervention capable of modifying the disease course. AIT has the potential to alter the natural history of allergic conditions by inducing immunological tolerance to specific allergens [8]. It is particularly indicated for patients experiencing moderate-to-severe symptoms inadequately controlled by standard pharmacotherapy or those seeking long-term prevention to reduce allergen sensitivity. In asthma management, AIT is unique as the only immune-modulating treatment currently recognized for targeting the foundational allergic mechanism, as endorsed by recent guidelines and authoritative recommendations such as those from the Global Initiative for Asthma (GINA 2025) [9]. GINA suggests that AIT can be considered an adjunctive therapy in both adult and pediatric patients with significant sensitization to aeroallergens, especially in cases where conventional treatments do not achieve optimal control [9]. Despite its promising role, the evidence supporting the efficacy of AIT in asthma management remains limited and somewhat inconclusive. There are ongoing debates about its impact on critical outcomes such as the frequency of exacerbations, improvements in lung function, and the potential for long-term disease modification [9]. More extensive, well-designed studies are necessary to establish definitive benefits, optimize patient selection criteria, and fully understand the therapeutic potential of AIT in asthma.

Our study aimed to evaluate the effectiveness of sublingual immunotherapy (SLIT) specifically in patients diagnosed with allergic asthma. We focused on whether SLIT could reduce the frequency and severity of asthma exacerbations and whether it might contribute to improvements in lung function parameters. Through this analysis, we hope to provide valuable evidence supporting the role of SLIT as a safe, effective, and potentially disease-modifying treatment option for individuals suffering from allergic asthma, contributing to the ongoing efforts to improve management strategies for this complex and impactful condition.

## 2. Materials and Methods

### 2.1. Study Design and Objectives

We conducted a retrospective study involving adult patients (≥18 years) affected by AA and AR who were followed at the Pulmonology Department of Trieste and the Allergy and Clinical Immunology Department of Pordenone between January 2022 and July 2025. Diagnosis of asthma was performed according to the Global Initiative for Asthma 2024 recommendations. The primary objective of this study was to assess the efficacy of SLIT in preventing exacerbations and to evaluate functional improvement in patients with AA after at least one year of treatment. Secondary objectives included determining the extent of clinical improvement through changes in the Asthma Control Test (ACT) score, evaluating a reduction in reliever medication use during the allergen season, and confirming the safety profile of the therapy.

### 2.2. Assessment of Allergen Sensitization

All patients were evaluated for sensitization to dust mites and grass pollen through measurement of specific IgE antibodies. For dust mites, IgE antibodies against the major allergens Der p 1, Der p 2, and Der p 23 were assessed. For grass pollen, IgE antibodies against the primary allergens Phl p 1 and Phl p 5 were investigated.

### 2.3. Sublingual Immunotherapy (SLIT) for Grass Pollen

Patients with grass pollen allergy received SLIT using either GRAZAX^®^ (ALK-Abelló A/S, headquartered in Hørsholm, Denmark) or ORALAIR^®^ (Stallergenes Greer International AG, headquartered in Baar, Switzerland). GRAZAX^®^ was administered as a single 75,000 SQ-T tablet once daily. ORALAIR^®^ was administered according to a step-up schedule: one 100-IR tablet on day 1, two 100-IR tablets on day 2, and one 300-IR tablet from day 3 onward. Both treatments were initiated four months prior to the anticipated start of the pollen season and continued until the end of the season to maximize therapeutic effectiveness.

### 2.4. Sublingual Immunotherapy (SLIT) for Dust Mites

Patients with dust mite allergy received SLIT using either Stallergenes Orylmyte^®^ (Stallergenes SAS, headquartered in Baar, Switzerland) or Lofarma LAIS^®^ Dermatophagoides (Lofarma S.p.A., headquartered in Milan, Italy). Orylmyte^®^ was administered using a step-up schedule: one 100-IR tablet on day 1, two 100-IR tablets on day 2, and one 300-IR tablet from day 3 onward. LAIS^®^ was administered according to the following regimen: ¼ tablet (1000 UA) on day 1, ½ tablet (1000 UA) on day 2, one full tablet (1000 UA) on day 3, and one 1000-UA tablet three times per week from day 4 onward.

### 2.5. Study Assessment

After a minimum of one year of SLIT, patients were evaluated for potential improvements in functional parameters and clinical outcomes related to both AR and bronchial asthma. Changes in reliever medication use and the occurrence of adverse events leading to treatment discontinuation were also recorded.

For patients with grass pollen allergy, functional assessments were restricted to the period of allergen exposure, defined as April to August; measurements obtained outside this timeframe were excluded from the analysis. This restriction was not applied to patients receiving SLIT for dust mite allergy, as exposure to these allergens is perennial.

### 2.6. Statistical Analysis

Normality of data was assessed by using the Shapiro–Wilk normality test. Categorical variables were summarized using frequencies and percentages, while continuous variables were expressed as mean ± standard deviation (SD) or median (interquartile range, IQR) according to their distribution. Associations between categorical variables were examined using the chi-square (χ^2^) test of independence. Particularly, χ^2^ test was applied to enhance differences and compare exacerbations, use of relievers more than twice a week, and ACT score > 20 before and after SLIT. Differences between continuous variables such as lung function tests (FEV1, FVC and FEV1/FVC) pre and post SLIT were examined through a paired *t*-test. A *p* < 0.05 was deemed statistically significant.

## 3. Results

A total of 24 patients (14 males and 10 females) with bronchial asthma and AR who had been on SLIT for at least one year were included in our study. Of these, 17 underwent SLIT for grass pollen and 7 for dust mites. Demographics and functional data of the enrolled subjects are presented in Table 1.

**Figure 1:**
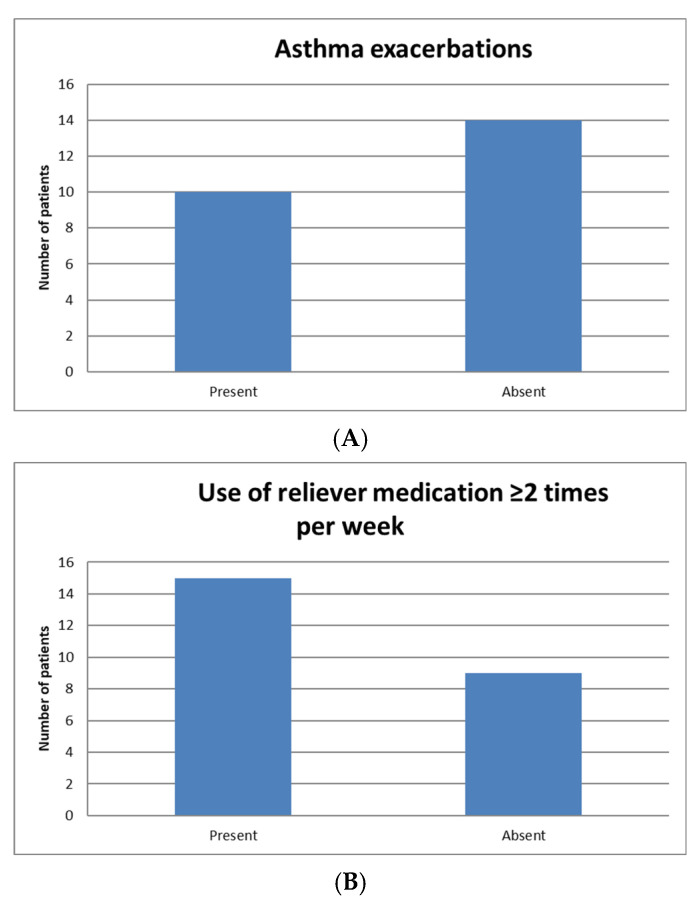

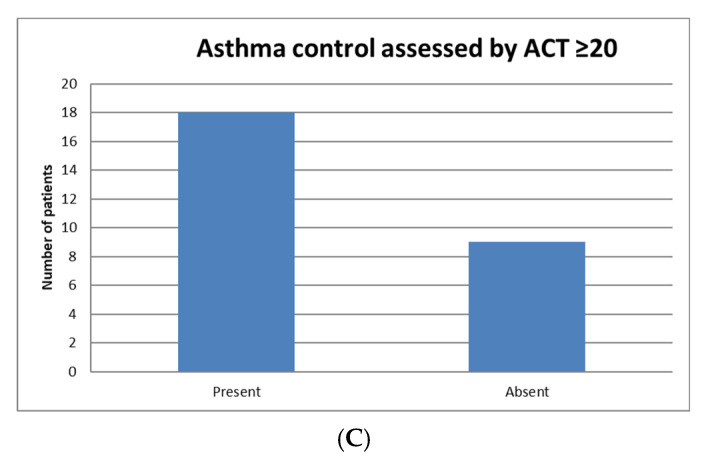
Distribution of patients by presence of asthma exacerbations (**A**), use of reliever medication ≥ 2 times per week (**B**), and asthma control assessed using the Asthma Control Test (ACT ≥ 20) (**C**). Data are expressed as number of patients. ACT: Asthma Control Test.

Only six patients (25%) reported incomplete symptom control (defined as an ACT score < 20), whereas all patients had experienced rhinitis symptoms prior to initiating SLIT. Regarding respiratory functional assessment, only one patient (4%) showed an obstructive ventilatory defect on pulmonary function testing. Overall, this patient group already exhibited good asthma control prior to initiating SLIT (only 25% had an ACT < 20), yet more than half of them (58%) had experienced at least one exacerbation in the year preceding treatment (Figure 1). In our patient cohort, 15 individuals (62.5%) used reliever medication at least twice per week during the allergen season, while the remaining nine reported less frequent use (Figure 1).

Following the initiation of SLIT, the study population showed improved clinical asthma control, with only one patient still presenting an ACT score < 20. This improvement, although clinically relevant, did not reach statistical significance (*p* = 0.07). All patients reported an improvement in rhinitis symptoms.

**Figure 2:**
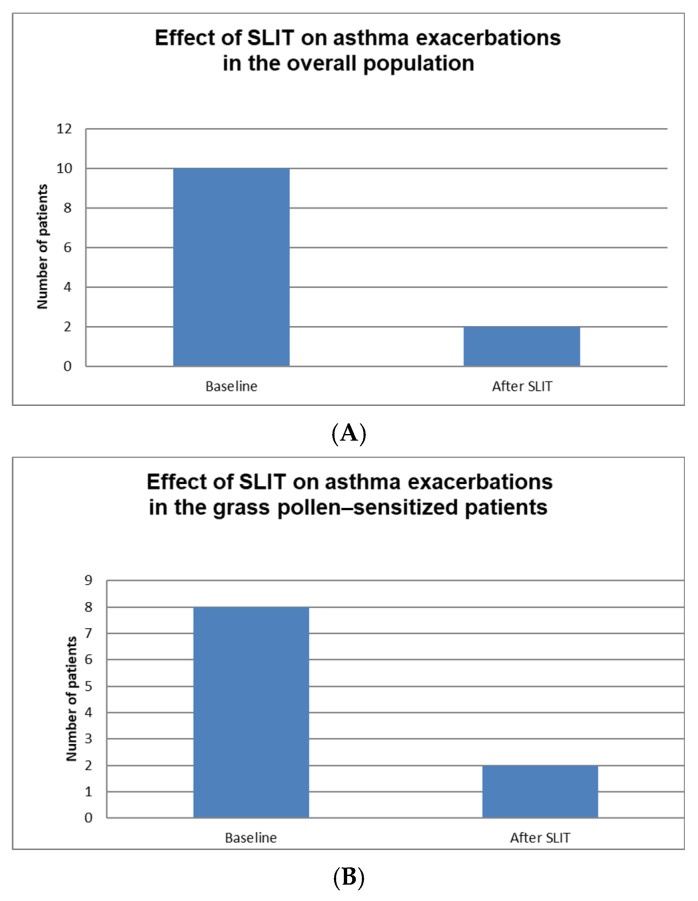
(**A**,**B**) Comparison of asthma exacerbations at baseline and after sublingual allergen immunotherapy (SLIT) in the overall population (**A**) and in patients with grass pollen sensitization (**B**). A statistically significant reduction in the number of patients experiencing asthma exacerbations was observed after treatment (*p* < 0.001). SLIT: sublingual allergen immunotherapy.

**Figure 3:**
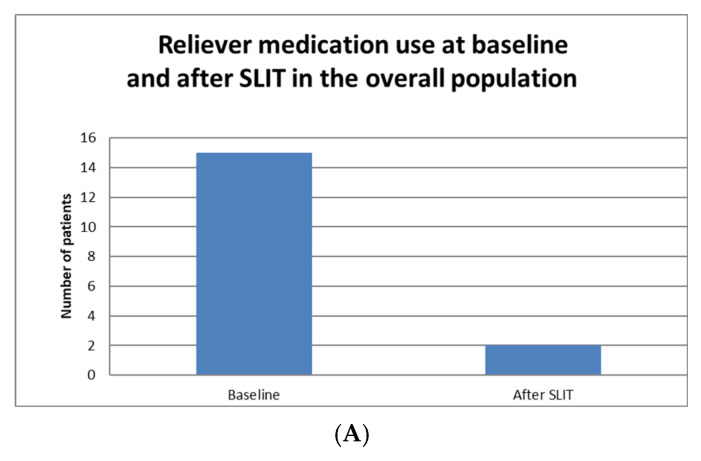

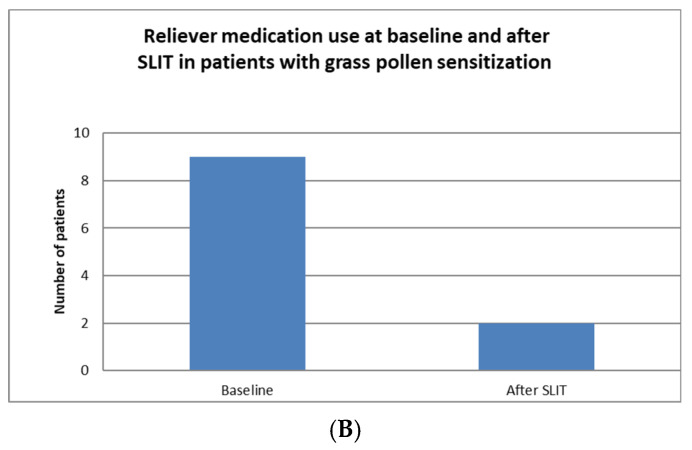
Comparison of reliever medication use at baseline and after sublingual allergen immunotherapy (SLIT) in the overall population (**A**) and in patients with grass pollen sensitization (**B**). A statistically significant reduction in frequent reliever use (≥2 times per week) was observed after treatment. SLIT: sublingual allergen immunotherapy.

A noteworthy finding was the reduction in the rate of exacerbations: only two patients (8%) of the ten who had previously experienced at least one exacerbation continued to report such events, whereas none of the patients who had reported no exacerbations during the pre-treatment period experienced any after initiating therapy (*p* < 0.01) (Figure 2). This finding was confirmed when considering exclusively the subgroup of patients undergoing SLIT for grass pollen allergy (Figure 3). Among the 17 patients evaluated, eight (47%) had experienced at least one exacerbation in the year preceding treatment initiation; of these, only two (11.7%) reported an exacerbation after starting therapy (*p* = 0.03).

Similarly, a reduction in the use of reliever medication during the allergic season was observed. Of the 15 patients who had previously used the reliever at least twice per week, only two continued to do so after one year of therapy. Moreover, none of the nine patients who had initially used it no more than once per week showed an increase in frequency of use (*p* < 0.001) (Figure 3).

The same trend was evident in the subgroup of patients receiving SLIT for grass pollen. Among the 17 patients examined, nine reported using the reliever at least twice per week during the pollen season prior to treatment. After at least one year of therapy, only two patients maintained this frequency, whereas none of the eight patients who had previously used it fewer than two times per week required an increased frequency of administration (*p* = 0.01).

Furthermore, after at least one year of SLIT, a significant improvement in asthma control was observed. The number of patients experiencing asthma exacerbations decreased markedly from 10 at baseline to 2 after treatment (*p* < 0.001). Similarly, the use of reliever medication more than twice per week was significantly reduced, from 15 patients at baseline to 2 after SLIT (*p* < 0.001). The number of patients with an ACT score ≥ 20 increased from 18 to 23, although this difference did not reach statistical significance (*p* = 0.07). Similarly, no statistically significant changes were observed in lung function parameters, including Forced expiratory volume at 1° second (FEV1) (3.45 ± 0.76 vs. 3.50 ± 0.90; *p* = 0.544), Forced vital capacity (FVC) (4.31 ± 1.02 vs. 4.50 ± 1.18; *p* = 0.177), and the FEV1/FVC ratio (80.04 ± 7.02 vs. 79.33 ± 7.19; *p* = 0.507). Additionally, the absence of significance in lung function parameters may be attributed to the preserved respiratory function of the patients at baseline.

## 4. Discussion

In this study, we aimed to evaluate the administration of sublingual immunotherapy (SLIT) in patients affected by allergic rhinitis (AR) and allergic asthma (AA).

It is important to note that the subgroup of patients receiving dust mite SLIT was small, which limits the strength and generalizability of these findings. Therefore, these results should be regarded as preliminary and exploratory, warranting further investigation in larger, prospective studies. It is well established that SLIT is recommended primarily for patients with AA when the disease is adequately controlled, as uncontrolled asthma is considered a recognized risk factor during allergen immunotherapy. Consequently, patients with an FEV_1_ value below 70% of the predicted or those who have experienced recent severe asthma exacerbations are generally deemed ineligible for initiating immunotherapy. These criteria are outlined in current guidelines to ensure patient safety and optimize treatment outcomes. The primary objective of our investigation was to explore whether SLIT might be associated with a reduction in the rate of asthma exacerbations and to assess its potential impact on respiratory function during periods of pollen exposure. It is important to clarify that, although some previous studies have suggested beneficial effects of SLIT on asthma exacerbations—particularly in the context of house dust mite immunotherapy—the evidence specifically regarding grass pollen–specific immunotherapy remains limited [10]. Our data, which included patients receiving SLIT for both house dust mite and grass pollen allergens, demonstrated a statistically significant reduction in asthma exacerbations within the study cohort (*p* < 0.01). These findings align with existing literature supporting the notion that SLIT may be associated with a decreased frequency of exacerbations [11]. However, it is crucial to interpret these results with caution, as the observational nature of the study precludes definitive conclusions about causality. In recent years, there has been an increasing trend in clinical practice toward using allergen-specific immunotherapy not only for isolated AR but also as a therapeutic strategy in AA and pollen-induced asthma. Such approaches have been linked to reductions in the rate of exacerbations and a decreased need for both maintenance and reliever medications [10,11]. In both adult and pediatric populations, these observations are particularly well documented for dust mite SLIT, where guidelines from the European Academy of Allergy and Clinical Immunology (EAACI) and systematic reviews such as the 2020 Cochrane review—citing studies by Virchow et al. (2016) and Wang et al. (2014)—have provided substantial evidence supporting efficacy [12,13,14,15]. Nonetheless, the amount of robust data available regarding grass pollen SLIT remains comparatively limited, partly due to the heterogeneity of study designs and populations. Recent guidelines from Germany on allergen immunotherapy in IgE-mediated allergic diseases have also acknowledged this gap, emphasizing the need for further research [16]. The primary study referenced in this context is a Danish epidemiological investigation involving patients with both perennial and seasonal AA, including those sensitized to grass pollen. The findings indicated that allergen immunotherapy significantly reduced the risk of exacerbations and lower respiratory tract infections in both seasonal and perennial forms of AA [17]. Within this framework, our study provides additional data on this specific aspect, suggesting that SLIT may have a role in reducing exacerbation frequency even among grass pollen–sensitized patients. An additional objective was to investigate whether these patients also experienced functional improvements—such as enhancements in spirometric parameters. However, no significant changes were observed in lung function measures (*p* = 0.544, *p* = 0.177, *p* = 0.507). These results could be influenced by several factors, including the baseline preservation of pulmonary function in our cohort and the relatively small sample size, which may limit the statistical power to detect subtle changes. Moreover, the lack of significant functional improvements aligns with previous findings that report heterogeneity in respiratory outcomes following SLIT, with some studies demonstrating variable evidence regarding its effectiveness in improving pulmonary function [18]. For instance, a retrospective Chinese study analyzing the clinical efficacy of AIT for Alternaria and Dermatophagoides farinae reported a significant increase in FEV_1_ after treatment [19]. Conversely, a study conducted at the University of Messina focusing on the effects of AIT in children with Parietaria-induced AA showed improvements in bronchial hyperreactivity as measured by methacholine challenge but did not observe significant changes in FEV_1_ [20]. These contrasting results underscore the complexity of assessing functional outcomes and suggest that patient-specific factors, disease phenotype, and the duration of therapy may influence the degree of functional improvement. Regarding the assessment of respiratory parameters during the pollen season—considered the period of greatest variability in asthma control—our findings are consistent with existing literature. In a randomized controlled trial by Marogna et al., which evaluated SLIT in polysensitized patients, significant clinical benefits were observed; however, functional respiratory parameters did not show equally marked changes [21]. Similarly, another study from the same group examined the effects of SLIT for birch pollen allergy, noting that functional improvements—including spirometric measures—began only after the second year of therapy, with the control group experiencing progressive functional decline [22]. These observations suggest that functional benefits may require longer treatment durations to manifest or may be more subtle than symptomatic improvements. One notable finding was the statistically significant reduction (*p* < 0.001) in the use of reliever medications during the pollen season, which further supports the potential clinical efficacy of SLIT in managing pollen-induced asthma exacerbations. Similar results have been reported in other studies, including a Japanese prospective observational study evaluating SLIT for Japanese cedar pollen allergy [23]. In a separate investigation, grass-sensitized patients were divided into three groups—continuous SLIT, pre-seasonal SLIT, and on-demand pharmacotherapy—with both SLIT groups experiencing reductions in reliever medication use compared to baseline and the control group, although no significant functional improvements were observed [20]. Concerning the secondary objectives, a marked clinical improvement was observed in patients undergoing SLIT, with most participants (75%) reporting good symptom control before therapy (as indicated by an Asthma Control Test score > 20). After at least one year of treatment, only one patient continued to report symptoms. While this suggests a positive trend, the lack of statistical significance (*p* = 0.07) likely reflects the limited sample size and the retrospective design. Importantly, the improved symptom control following immunotherapy initiation has been widely documented, particularly in patients receiving SLIT for dust mite allergy [24,25]. The safety profile of SLIT was excellent in our cohort, with no patients requiring discontinuation due to adverse effects. This observation reinforces current guidelines from the American Refractory Asthma and the European Academy of Allergy and Clinical Immunology (EAACI), as well as recent reviews emphasizing the favorable safety profile of SLIT tablet formulations [26]. Such safety data support the potential for allergen immunotherapy to modify the natural history of allergic diseases, possibly preventing AR from progressing to AA under continuous allergen exposure. Although this remains an intriguing hypothesis, further studies are necessary to confirm whether SLIT can alter disease progression, particularly in pollen-induced asthma or in atopic patients with established asthma [27]. Theoretical considerations suggest that removing the stimulus that drives Th2-mediated inflammation might prevent subsequent airway remodeling and disease severity escalation; however, definitive evidence supporting this mechanism is currently lacking.

Our study has several limitations that should be acknowledged. First, its retrospective, observational design—based on the review of medical records—introduces potential selection bias and precludes definitive causal inferences between SLIT initiation and the observed clinical outcomes. The relatively small sample size, especially in the subgroup receiving SLIT for house dust mite allergens, limits the statistical power and may have contributed to the absence of significant findings in some functional measures. Given the retrospective observational nature of this study and the lack of a control group, these findings should be interpreted as associations rather than evidence of a causal therapeutic effect. Additionally, the study population generally exhibited good disease control at baseline, which could lead to a ceiling effect, reducing the likelihood of detecting meaningful improvements. We recognize that observational studies carry inherent limitations and future randomized controlled trials are necessary to establish definitive efficacy. While our study did not observe significant changes in lung function parameters, it is important to recognize that reductions in exacerbation frequency and medication use are highly relevant clinical outcomes. Recent evidence suggests that in patients with mild or well-controlled asthma, preventing exacerbations may be more meaningful than modest improvements in spirometric measures.

## 5. Conclusions

Given the retrospective observational design and the limited sample size—particularly within the subgroup of patients treated with dust mite SLIT—our findings should be interpreted with caution. The observed associations between SLIT and improvements in clinical outcomes, such as symptom control and a reduction in exacerbations, appear more consistent in patients undergoing grass pollen SLIT, for which an acknowledgment is made that spirometric measures may remain stable in well-controlled patients, and that a reduction in exacerbations and medication use are valuable markers of improved disease control. Conversely, the findings related to dust mite SLIT are exploratory and based on a smaller cohort, which limits their generalizability and warrants careful consideration before extrapolation. No significant effects on pulmonary function were observed across the entire cohort. Future prospective, controlled studies involving larger and more diverse populations are essential to more definitively assess the efficacy and potential disease-modulating effects of SLIT, especially in patients sensitized to dust mites. Such research will help clarify the role of SLIT in improving respiratory outcomes and guide its application in clinical practice.

## Figures and Tables

**Table 1:** Demographics, functional, and immunological characteristics of the study population.

Demographics	*N* = 24
Age, years (mean ± SD)	35.4 ± 12.3
Gender (M/F) n, %	14 (58.3%)/10 (41.6%)
Lung function	
FEV1 pre, L (mean ± SD)	3.45 ± 0.76
FEV1 post, L (mean ± SD)	3.50 ± 0.90
FVC pre, L (mean ± SD)	4.31 ± 1.01
FVC post, L (mean ± SD)	4.53 ± 1.18
FEV1/FVC pre, (mean ± SD)	80.04 ± 7.02
FEV1/FVC post, (mean ± SD)	79.33 ± 7.19
Immunological characteristics	
Grass Pollen P1 (kU/L), n (mean ± SD)	16 (11.32 ± 10)
Grass Pollen P5 (kU/L), n (mean ± SD)	16 (5.96 ± 7.7)
Dust Mites DERP 1 (kU/L), n (mean ± SD)	5 (11 ± 10.8)
Dust Mites DERP 2 (kU/L), n (mean ± SD)	6 (9.26 ± 10.2)
Dust Mites DERP 23 (kU/L), n (mean ± SD)	4 (3.21 ± 2.17)

## Data Availability

Deidentified participant data are available upon reasonable request, subject to review and approval by the local Ethics Committee. Interested researchers may submit amotivated request to the Corresponding Author, who will forward it to the Ethics Committee for evaluation.
